# Uncovering a population of gravitational lens galaxies with magnified standard candle SN Zwicky

**DOI:** 10.1038/s41550-023-01981-3

**Published:** 2023-06-12

**Authors:** Ariel Goobar, Joel Johansson, Steve Schulze, Nikki Arendse, Ana Sagués Carracedo, Suhail Dhawan, Edvard Mörtsell, Christoffer Fremling, Lin Yan, Daniel Perley, Jesper Sollerman, Rémy Joseph, K-Ryan Hinds, William Meynardie, Igor Andreoni, Eric Bellm, Josh Bloom, Thomas E. Collett, Andrew Drake, Matthew Graham, Mansi Kasliwal, Shri R. Kulkarni, Cameron Lemon, Adam A. Miller, James D. Neill, Jakob Nordin, Justin Pierel, Johan Richard, Reed Riddle, Mickael Rigault, Ben Rusholme, Yashvi Sharma, Robert Stein, Gabrielle Stewart, Alice Townsend, Jozsef Vinko, J. Craig Wheeler, Avery Wold

**Affiliations:** 1 The Oskar Klein Centre, Department of Physics, Stockholm University, AlbaNova University Center, Stockholm, Sweden; 2 Institute of Astronomy and Kavli Institute for Cosmology, University of Cambridge, Cambridge, UK; 3 Cahill Center for Astrophysics, California Institute of Technology, Pasadena, CA USA; 4 Astrophysics Research Institute, Liverpool John Moores University, Liverpool, UK; 5 The Oskar Klein Centre, Department of Astronomy, Stockholm University, AlbaNova University Center, Stockholm, Sweden; 6 Joint Space-Science Institute, University of Maryland, College Park, MD USA; 7 Department of Astronomy, University of Maryland, College Park, MD USA; 8 Astrophysics Science Division, NASA Goddard Space Flight Center, Greenbelt, MD USA; 9 DIRAC Institute, Department of Astronomy, University of Washington, Seattle, WA USA; 10 Department of Astronomy, University of California, Berkeley, CA USA; 11 Institute of Cosmology and Gravitation, University of Portsmouth, Portsmouth, UK; 12 Institute of Physics, Laboratoire d’Astrophysique, Ecole Polytechnique Fédérale de Lausanne (EPFL), Observatoire de Sauverny, Versoix, CH Switzerland; 13 Department of Physics and Astronomy, Northwestern University, Evanston, IL USA; 14 Center for Interdisciplinary Exploration and Research in Astrophysics (CIERA), Northwestern University, Evanston, IL USA; 15 Institut fur Physik, Humboldt-Universität zu Berlin, Berlin, Germany; 16 Space Telescope Science Institute, Baltimore, MD USA; 17 Université Lyon 1, ENS de Lyon, CNRS, Centre de Recherche Astrophysique de Lyon UMR5574, Saint-Genis-Laval, France; 18 Caltech Optical Observatories, California Institute of Technology, Pasadena, CA USA; 19 Université de Lyon, Université Claude Bernard Lyon 1, CNRS/IN2P3, IP2I Lyon, Villeurbanne, France; 20 IPAC, California Institute of Technology, Pasadena, CA USA; 21 Department of Astronomy, University of Texas at Austin, Austin, TX USA; 22 CSFK, Konkoly Observatory, Budapest, Hungary

**Keywords:** Time-domain astronomy, General relativity and gravity, Cosmology

## Abstract

Detecting gravitationally lensed supernovae is among the biggest challenges in astronomy. It involves a combination of two very rare phenomena: catching the transient signal of a stellar explosion in a distant galaxy and observing it through a nearly perfectly aligned foreground galaxy that deflects light towards the observer. Here we describe how high-cadence optical observations with the Zwicky Transient Facility, with its unparalleled large field of view, led to the detection of a multiply imaged type Ia supernova, SN Zwicky, also known as SN 2022qmx. Magnified nearly 25-fold, the system was found thanks to the standard candle nature of type Ia supernovae. High-spatial-resolution imaging with the Keck telescope resolved four images of the supernova with very small angular separation, corresponding to an Einstein radius of only *θ*_E_ = 0.167″ and almost identical arrival times. The small *θ*_E_ and faintness of the lensing galaxy are very unusual, highlighting the importance of supernovae to fully characterize the properties of galaxy-scale gravitational lenses, including the impact of galaxy substructures.

## Abstract (web summary)

SN 2022qmx, dubbed SN Zwicky, is a rare, gravitationally lensed type Ia supernova, magnified by a factor of 25, discovered by the Zwicky Transient Facility. Follow-up Keck observations reveal four multiple images with unusually small separation.

## Main

Our understanding of gravitational lensing due to the curvature of spacetime, and the analogy with the deflection of light in optics, dates back to the work of Einstein in 1936^1^. In this pioneering work he considered the case where both the lens and the magnified background source were stars in our Galaxy. Einstein concluded that the deflection angles were too small to be resolved with astronomical instruments. It was Zwicky^2^ who one year later pointed out that, if the source was extragalactic, entire galaxies or clusters of galaxies could be considered as gravitational deflectors. Hence, the image separation between multiple images of the source could be large enough to be resolved by astronomical facilities, as the size of the image separation scales with the lens mass and distance as the Einstein radius, ${\theta }_{\rm{E}}\approx 0.{9}^{{\prime\prime} }{\left(\frac{{M}_{\rm{l}}}{1{0}^{11}{M}_{\odot }}\right)}^{\frac{1}{2}}{\left(\frac{{D}_{\rm{s}}}{{{1\,{\rm{Gpc}}}}}\right)}^{-\frac{1}{2}}{\left(\frac{{D}_{\rm{ls}}}{{D}_{\rm{l}}}\right)}^{\frac{1}{2}}$, where *M*_⊙_ is the mass of the Sun, *M*_l_ and *D*_l_ are the lensing mass and lens angular size distance and *D*_s_ and *D*_ls_ are the distances from the observer to the source and between the lens and the source, respectively.

## Strongly lensed supernovae in the era of wide-field time-domain surveys

Besides the many observations of lensed galaxies and quasars, the feasibility of observing strong gravitational lensing of explosive transients in the distant universe has only been demonstrated in recent years (refs. ^3–5^ and references therein). PS1-10afx was the first highly magnified type Ia supernova (SN Ia) discovered. However, the lensing interpretation was made three years after the explosion^6,7^; by then the SN was too faint to resolve multiple images. Since then, the use of wide-field optical cameras in robotic telescopes at the Palomar Observatory has led to notable breakthroughs. In ref. ^8^ we reported the discovery of a multiply imaged SN Ia, iPTF16geu (SN 2016geu), by the intermediate Palomar Transient Factory (iPTF), a time-domain survey using a 7.3°^2^ camera on the P48 (1.2 m) telescope from 2013 to 2017. In 2018, a new camera was installed^9^ with a field of view of 47°^2^. The project, known as the Zwicky Transient Facility (ZTF)^10,11^, has been monitoring the northern sky with a 2–3 d cadence in at least two optical filters for the past four years^12^. The very large sky coverage makes ZTF well suited to search for rare phenomena, such as gravitational lensing of supernovae. On the other hand, the distance (redshift) probed by ZTF is limited by the relatively small mirror of the telescope, light pollution, non-optimal atmospheric conditions and only having three optical filters at the P48 telescope. Furthermore, ZTF typically obtains an image quality (angular resolution) of 2″ full-width at half-maximum (FWHM), and the camera has relatively large 1″ pixels. Hence, in most instances, it is practically impossible to spatially resolve multiple-image systems with ZTF. Instead, the search for lensed sources makes use of the standard candle nature of type Ia supernovae, that is, they have nearly identical peak luminosities. These explosions are used as accurate distance estimators in cosmology, which led to the discovery of the accelerated expansion of the universe (ref. ^13^ and references therein).

In addition to an imaging survey telescope, ZTF has access to a low-spectral-resolution integral-field spectrograph, the SED Machine (SEDM)^14^, on the neighbouring 1.5 m telescope at Palomar (P60), used to spectroscopically classify about ten supernovae every night as part of the Bright Transient Survey (BTS), where transients brighter than 19 mag are classified within timescales of a few days, aiming to obtain >95% spectroscopic completeness to 18.5 mag or brighter^15^. Besides providing the classification of the transients, the SEDM spectrum is used to measure the SN redshift.

## The discovery of SN Zwicky

**Fig. 1:**
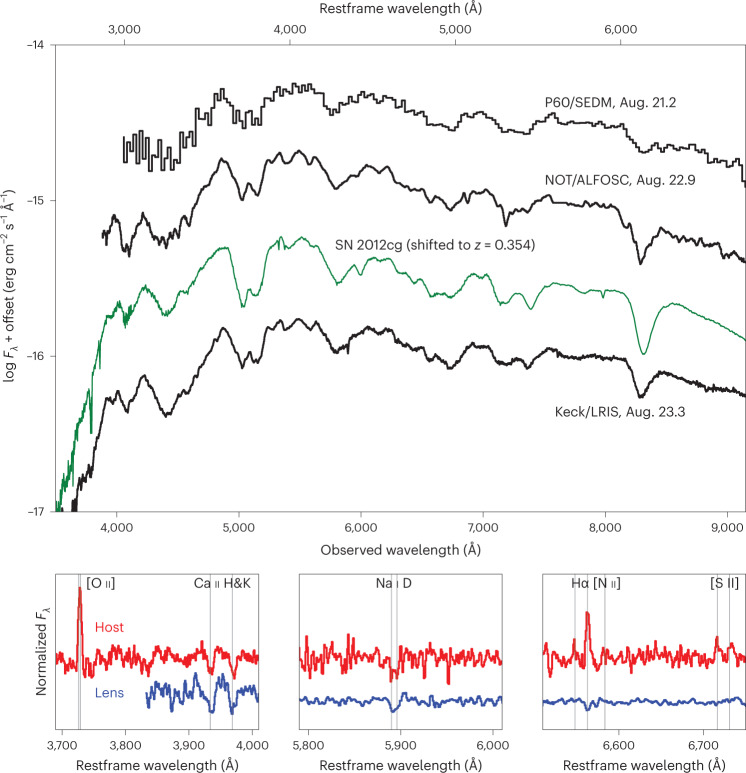
Spectroscopic identification of SN Zwicky as an SN Ia and redshift measurements of the SN host galaxy and the intervening lensing galaxy. The SN spectra obtained with the P60, Nordic Optical Telescope (NOT) and Keck telescopes (black lines) are best fitted by a normal SN Ia spectral template. The green line shows a comparison with the nearby type Ia SN 2012cg^69^ at a similar restframe phase, redshifted to *z* = 0.354. The SN flux peaked on 2022 August 17. The bottom panels show a zoomed-in view of a VLT/MUSE spectrum from 2022 September 30, displaying narrow absorption and emission lines, from which precise redshifts of the lens (*z* = 0.2262) and host (*z* = 0.3544) galaxies were determined. [O ii], Ca ii H and K, Na i D, Hα, [N ii] and [S ii] lines can be seen at the restframe of the lens (blue lines) and host (red lines) galaxies. ALFOSC, Alhambra Faint Object Spectrograph and Camera.

Lensed system candidates are selected by ZTF for further spectroscopic screening when an SN Ia is found at a redshift above *z* = 0.2, where there is essentially negligible sensitivity for detection in the BTS, unless the SN is greatly magnified by an intervening deflector. This was the case for SN Zwicky (also known as ZTF22aaylnhq and SN 2022qmx), located at right ascension 17 h 35 min 44.32 s and declination 4° 49′ 56.90″ (J2000.0), where an SEDM spectrum from 2022 August 21 showed it to be an SN Ia at *z* = 0.35, as shown in Fig. 1. At that point we alerted the SN community to the discovery of a lensed SN Ia^16^.

Spectroscopic observations following the time evolution of the SN were carried out using multiple facilities: the 2.56 m Nordic Optical Telescope in the Canary Islands, the Keck observatory in Hawaii, the 11 m Hobby–Eberly Telescope at the McDonald Observatory in Texas and the European Southern Observatory (ESO)’s 8 m Very Large Telescope (VLT) at the Paranal Observatory in Chile. In particular, multiple narrow emission and absorption lines of the SN host galaxy were found with the Low Resolution Imaging Spectrometer (LRIS)/Keck and the Multi-Unit Spectroscopic Explorer (MUSE)/VLT, refining the source redshift to *z* = 0.3544, as shown in the bottom panels of Fig. 1. As the SN faded and the foreground galaxy spectral energy distribution became more prominent, the Ca ii doublet *λλ*3,933, 3,968 was found in absorption lines redshifted to *z* = 0.22615, the location of the deflecting galaxy.

## Follow-up observations

**Fig. 2:**
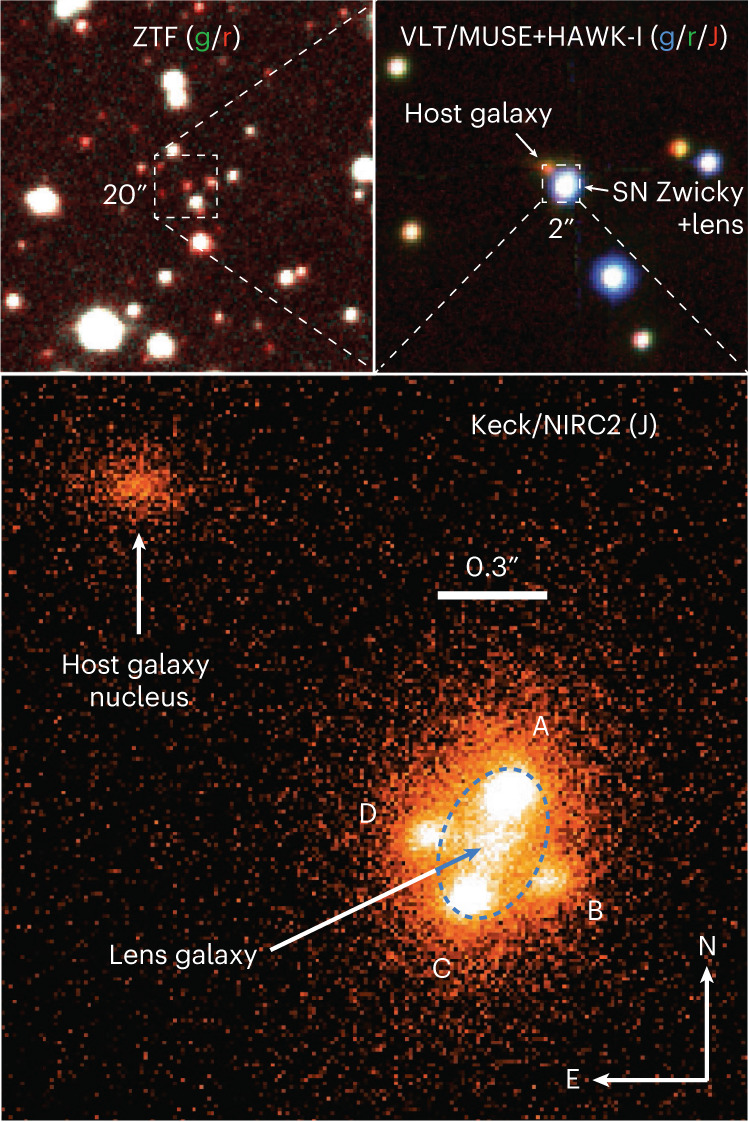
Image of the field of SN Zwicky using pre-explosion g- and r-band images from ZTF. Top left: a 2′ × 2′ section of the ZTF g- and r-band pre-SN images (FWHM 2.3″), centred on the location of SN Zwicky. Top right: a zoomed-in composite image of SN Zwicky using adaptive optics (AO)-enhanced VLT MUSE (g/r-band) and HAWK-I (J-band) observations on September 2 and 4 (FWHM ~0.4″). Bottom: a 2″ × 2″ portion of the Keck/NIRC2 LGSAO J-band image (FWHM 0.09″), resolving the four multiple images of SN Zwicky (labelled A, B, C, D). The blue dashed ellipse shows the critical line of the lens, corresponding to the inferred *θ*_E_ = 0.167″ (0.6 kpc at *z* = 0.2262), enclosing the lens *M* = (7.82 ± 0.06) × 10^9^ *M*_⊙_. The host galaxy nucleus is located 1.4″ to the northeast of the lens, implying that SN Zwicky exploded at a projected distance of 7 kpc from the centre of its host galaxy.

The discovery from ZTF was also followed up with high-spatial-resolution instruments. Observations with laser guide star enhanced seeing at the VLT with the High Acuity Wide-field K-band Imager (HAWK-I) imaging camera in the near infrared, and optical spectrophotometry with MUSE, reduced the point spread function (PSF) width to about 0.4″. However, this was still not enough to resolve the system. On 2022 September 15, multiple images of the system were resolved at near-infrared wavelengths at the Keck telescope, using the Laser Guide Star Aided Adaptive Optics (LGSAO) with Near-IR Camera 2 (NIRC2)^17^, yielding an image quality of 0.09″ FWHM in the J band centred at 1.2 μm, shown in Fig. 2, where the four SN images are labelled A–D.

On September 21, following our announcement of the discovery^16^, a previously approved programme aimed to target lensed supernovae by the LensWatch collaboration resolved the multiple images of SN Zwicky using the optical filters F475W, F625W and F814W (where the names correspond to the approximate location of the central wavelength in nanometres) on the UVIS/WFC3 camera on the Hubble Space Telescope (HST)^18^. A detailed description of the HST observations of SN Zwicky is presented in ref. ^19^.

## Results

**Fig. 3:**
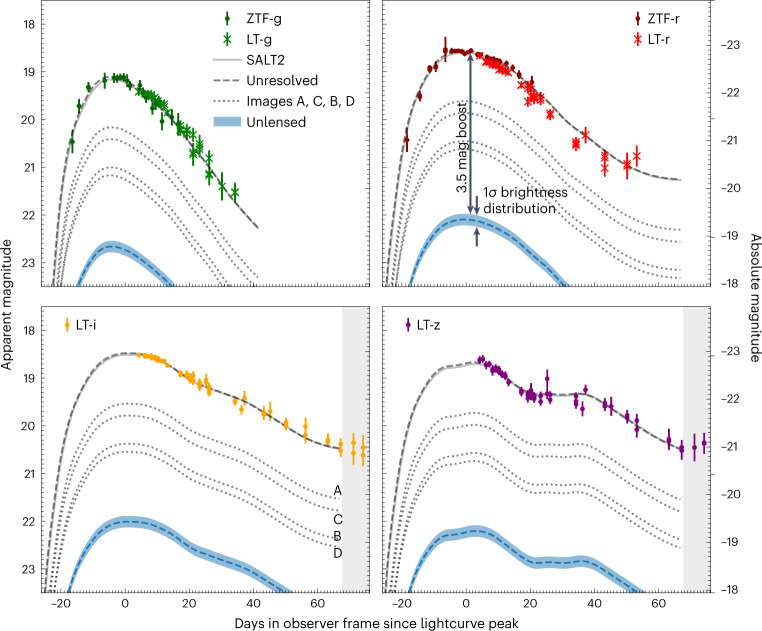
Multicolour lightcurve of SN Zwicky showing that the supernova is 3.5 mag brighter than an unlensed SN at the same redshift. The magnitudes are measured with respect to time of maximum light (modified Julian Date 59808.67, corresponding to 2022 August 17) in ZTF g and r and Liverpool Telescope (LT) g, r, i, z filters. The solid lines show the SALT2^20^ model with the best fit to the spatially unresolved data. The blue dashed lines indicate the expected lightcurves at *z* = 0.354 (without lensing), where the bands represent the s.d. of the brightness distribution for type Ia supernovae. To fit the observed lightcurves, a brightness increase corresponding to 3.5 mag is required. Also shown, as dotted lines, are the modelled individual lightcurves for the four SN images A–D. The flux ratios were measured from the NIRC2 data in Fig. 2. From these lightcurves we extract the time delays between the images, all in units of days, Δ*t*_AB_ = −0.4 ± 2.9, Δ*t*_AC_ = −0.1 ± 2.3 and Δ*t*_AD_ = −0.1 ± 2.7, as described in Supplementary Information. The shaded areas in the lower panels indicate the regions with data outside the phase range where the SALT2 model is defined, and therefore excluded from the lightcurve fit analysis. The error bars correspond to 1 s.d.

Figure 3 shows the unresolved photometric ground-based observations collected at P48 and the Liverpool Telescope in the Canary Islands between 2022 August 1 and October 30. These were used to estimate the peak flux and lightcurve properties of the SN with the SALT2 lightcurve fitting tool^20^, including corrections for lightcurve shape and colour excess given the SN redshift, as well as the extinction in the Milky Way in the direction of the SN . Furthermore, the four resolved SN images were used to explore the possibility of additional reddening by dust in the lensing galaxy. Unaccounted-for dimming of light would lead to an underestimation of the lensing amplification. The HST and NIRC2 observations for each SN image were compared with the SN Ia spectral template from ref. ^21^, allowing for possible differential dust extinction in the lens following the reddening law in ref. ^22^.

No evidence for differential extinction between the different images was found. The lightcurve fit model included the four individual SN images, described by the SALT2 model with arbitrary time delays, but otherwise sharing the same lightcurve shape and colour parameters, *x*_1_ and *c*. The time delays were constrained by a prior on the image flux ratios from the NIRC2 observations shown in Fig. 2. We find Δ*t*_AB_ = −0.4 ± 2.9, Δ*t*_AC_ = −0.1 ± 2.3 and Δ*t*_AD_ = −0.1 ± 2.7 (in units of days), where the indices A–D refer to the SN images in Fig. 2. The resulting lightcurve fit is shown in Fig. 3 and compared with the SALT2 model. The small time delays are also consistent with the spectra of SN Zwicky shown in Fig. 1 being at a single SN phase.

The fitted SN model parameters are *x*_1_ = 1.083 ± 0.094 and *c* = −0.007 ± 0.007. The lack of colour excess confirms that differential extinction is negligible. Since the lightcurve parameter errors do not include the model covariance, we conservatively add the SALT2 model error floor of *σ*(*x*_1_) = 0.1 and *σ*(*c*) = 0.027 mag (ref. ^23^) in quadrature to the fit errors. Using the inferred apparent magnitude and the SALT2 parameters above, we find a total magnification of Δ*m* = −3.44 ± 0.14 mag, assuming standard cosmological parameters from ref. ^24^ and a restframe B-band SN Ia peak absolute magnitude of −19.4 mag for the average SN Ia lightcurve width and colour, and intrinsic brightness scatter of 0.1 mag. Since the inferred stellar mass of the host galaxy is *M*_★_ ≲ 10^10^ *M*_⊙_, mass-step corrections for the SN Ia absolute magnitude as suggested in ref. ^25^ are not required. In summary, we find that, including the four images, SN Zwicky is 23.7 ± 3.2 times brighter than the observed flux of normal type Ia supernovae at the same redshift, after applying colour and lightcurve shape corrections.

The Keck NIRC2 J-band image was used to obtain a lens model to account for the observed SN image positions, irrespective of their fluxes. Assuming a singular isothermal ellipsoid^26,27^ for the lens potential, we report an ellipticity *ϵ*_e_ = 0.35 ± 0.01 and *θ*_E_ = 0.1670 ± 0.0006″. The mass enclosed within the ellipse with semi-major axis 0.78 kpc and semi-minor axis 0.51 kpc is *M* = (7.82 ± 0.06) × 10^9^ *M*_⊙_. The lens model predictions for the time delays are in excellent agreement with the fitted values from the lightcurves in Fig. 3. Further details regarding the lens modelling are presented in Methods.

Interestingly, the individual image magnifications predicted for SN Zwicky by the smooth macro lens model are inconsistent with the observed flux ratios. According to the lensing model, the observed fluxes of the SN images A and C are factors of >4 and >2 too large, respectively, compared with images B and D. Given the small time delays, this discrepancy cannot be accounted by different phases between the SN images. Other explanations need to be considered: for example, excess magnification and demagnification from milli- and microlensing effects arising from stars and substructure in the lens galaxy^28,29^. Since microlensing effects are capable of perturbing magnifications without altering image locations, these were also put forward to explain the observed flux ratios of iPTF16geu^30^, displaying differences between the observed and modelled image flux ratios of similar magnitudes. Probing microlensing in these central regions opens a new window to directly measure the central stellar initial mass function^31^ and test claims that the initial mass function may be heavier in the centres of galaxies^32^. As detailed in Methods, the lack of time dependence in the image flux ratios, or anomalous variation in the unresolved lightcurves, gives a lower limit for the substructure masses of 0.02 *M*_⊙_, if the discrepancy from the smooth lens model is caused by microlensing. From the lack of further image splitting of the four individual SN images, we infer an upper limit for the substructure mass of 3 × 10^7^ *M*_⊙_.

To check the impact of added macro lens model complexity, we have studied cases where the lens mass distribution is modelled with two matter components; one where the surface mass density follows the lens light distribution (with an arbitrary mass-to-light ratio, possibly interpreted as a baryonic mass component), and a second one introducing a dark matter halo with additional flexibility on density profiles. In spite of the added extra complexity, the quality of the fit to the SN image positions does not improve, and induces shifts in the predicted flux ratios only below 5%, that is, very small in comparison with the observed flux ratio anomalies.

**Fig. 4:**
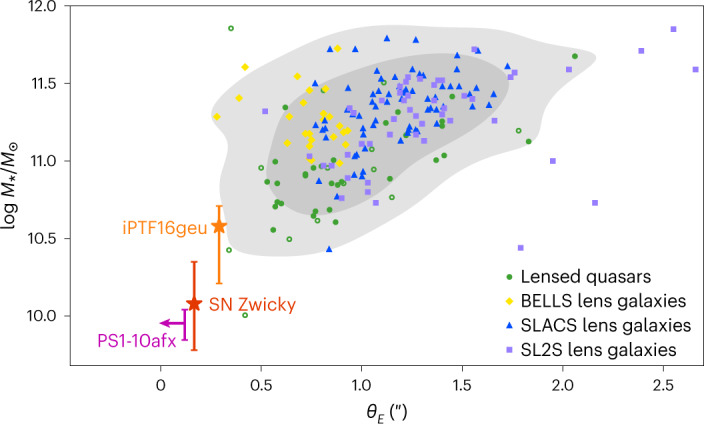
Stellar mass versus Einstein radius for lens galaxies discovered in galaxy surveys, demonstrating that SN Zwicky, iPTF16geu and the unresolved lensed supernova PS1-10afx point to a poorly known population of small-image-separation lensing systems. Strongly lensed galaxy systems are represented by yellow diamonds for the BOSS Emission-Line Lens Survey (BELLS) sample^70^, blue triangles for Sloan Lens ACS (SLACS) lenses^71^ and purple squares for SL2S lenses^72^. The green circles correspond to lensed quasars^73^, of which the filled circles have been detected optically and the open circles through radio emission. The shaded grey contours show the 90% and 68% confidence levels for the full sample of 155 lensed galaxies and 45 lensed quasars. The lensed supernova data are presented as median values ± 1 s.d. For the unresolved lensed supernova PS1-10afx, only an upper limit of the Einstein radius is available^7^. The stellar mass derivation for SN Zwicky is detailed in Methods.

The demonstrated ability to discover multiply imaged supernovae makes it feasible to accomplish Refsdal’s pioneering proposal ^33^ to use time delays for strongly lensed supernovae to measure the Hubble constant. This will require systems with time delays of several days, that is, longer than for SN Zwicky. The small physical scale of the lens probed by SN Zwicky, as well as iPTF16geu^8^, make these supernovae unique tracers for uncovering a population of systems that otherwise would remain undetected, as shown in Fig. 4, probing the mass distribution of the central, densest, regions of lensing galaxies. Both of these multiply imaged type Ia supernovae were identified without preselections on, for example, association with bright galaxies or clusters, emphasizing the importance of untargeted surveys for unexpected discoveries.

## Methods

### Supernova survey and follow-up

The ZTF has been monitoring the transient sky at optical wavelengths since 2018^9–11,34^. SN Zwicky^16^ was discovered under the BTS programme^15^. The first detection of the SN was in a ZTF g-band image from 2022 August 1. It was saved to the BTS as an SN candidate by on-duty scanners on August 3^35^ and subsequently assigned to the queue for spectroscopic follow-up with SEDM mounted on P60 under standard BTS protocols. The SEDM spectrum, obtained on August 21^36^, shows an excellent match to a normal SN Ia at a redshift of *z* = 0.35 close to maximum light. The redshift and spectral classification were confirmed with a higher-resolution spectrum obtained at the Nordic Optical Telescope in La Palma on the following night. We followed up SN Zwicky with P48 in the g and r bands. For our analysis we use the forced photometry provided by the Image Processing and Analysis Center as detailed in ref. ^34^. Observations in the Sloan Digital Sky Survey g, r, i and z filters were taken with the IO:O optical imager on the Liverpool Telescope^37^. The Liverpool Telescope photometric data are processed with custom data-reduction and image-subtraction software (K. Taggart et al., manuscript in preparation). Image subtraction is performed using the Panoramic Survey Telescope and Rapid Response System 1 (Pan-STARRS1) reference image. Images were stacked using SWarp to combine multiple exposures where required. The photometry is measured using a PSF fitting methodology relative to Pan-STARRS1 standards and is based on techniques in ref. ^38^.

### Lightcurve fit, magnification and time-delay inference

We used the publicly available, Python-based software SNTD^39^ for inferring the restframe B-peak magnitude, the lightcurve shape and colour SN Ia SALT2 parameters^23^ and the time delays between the SN images. Data points with ≥3*σ* detections from the g and r filters from P48 and the g, r, i, z filters from the Liverpool Telescope were included in the fit. We adopted an iterative procedure in two steps. First, all the data were used. Next, only data points in the range where the SALT2 model is defined, that is, −20 to +50 d, were kept for the second iteration. The final lightcurve fit parameters were derived from data in this phase range.

We estimated the time delays, that is, the relative phase between the SN images, by fitting the unresolved ground-based flux data with the model that includes the flux contributions from the four sets of SN lightcurves, F^*j*^(*t*, *λ*), each one with its own time of B-band maximum, ${t}_{0}^{j}$. The fit is constrained by imposing a prior on the image ratios at the date of the Keck/NIRC2 observations, reported in Supplementary Table 3. We allow the lightcurve fit parameters to vary within the ranges *x*_1_ = [−3.0, 3.0], *c* = [−0.3, 0.3], but assume them to be the same for all four images. This is an excellent approximation in the absence of appreciable differential reddening in the lensing galaxy, confirmed by the result of the fit, *c* = −0.01 ± 0.01, consistent with no colour excess.

Galactic extinction was included in the model, adopting *E*(*B* − *V*)_MW_ = 0.1558 mag, based on the extinction maps in ref. ^40^. We used the wavelength dependence from the dust from ref. ^22^ and the measured mean value of the total to the Galactic selection extinction ratio, *R*_*V*_ = 3.1. From the location of the fitted restframe B-band peak luminosity in the SALT2 model for the summed fluxes we inferred the time of maximum for SN Zwicky, *t*_0_ = 59808.67 ± 0.19, corresponding to 2022 August 17. The total lensing magnification, *μ* = 24.3 ± 2.7, was calculated after standardizing the SN Ia fitted B-band peak magnitude with the standard SALT2 lightcurve shape and colour correction parameters, (*α*, *β*) = (0.14, 3.1). Throughout the analysis of SN Zwicky, we adopted a flat *Λ* cold dark matter cosmological model with *H*_0_ = 67.4 km s^−1^ Mpc^−1^ and *Ω*_M_ = 0.315 (ref. ^24^).

The uncertainties we report for the time delays account for parameter degeneracies in the fit. Corner plots with posteriors for the relative time delays of B, C and D with respect to A are illustrated in Supplementary Fig. 1. The unresolved data used for this analysis are available via WISeREP at https://www.wiserep.org/object/21343. It should be emphasized that the lightcurve and time-delay fits were carried out completely independently from the lens modelling.

### Spectroscopic follow-up

The first classification spectrum of SN Zwicky was obtained with the integral field unit on the SEDM on 2022 August 21. The data were reduced using a custom integral field unit pipeline developed for the instrument^41,42^. Flux calibration and correction of telluric bands were carried out using a standard star taken at a similar airmass. The details of all spectroscopic observations are listed in Supplementary Table 1.

We obtained three epochs of spectroscopy between 2022 August 21 and September 11 with ALFOSC on the 2.56 m Nordic Optical Telescope at the Observatorio del Roque de los Muchachos in La Palma (Spain) (see http://www.not.iac.es/instruments/alfosc for further information). Observations were taken using grism 4, providing wavelength coverage over most of the optical spectral range (typically 3,700–9,600 Å). Reduction and calibration were performed using PypeIt version 1.8.1^43,44^.

We obtained three epochs of spectroscopy between 2022 August 22 and October 19 with LRIS on the Keck I 10 m telescope^45^. All spectra were reduced and extracted with LPipe^46^.

### Adaptive optics observations

#### Observations with the VLT

##### HAWK-I observations and data reduction

We obtained seven epochs of near infrared in YJHK between 2022 August 23 and September 30 with HAWK-I^47–49^ at the ESO VLT at the Paranal Observatory (Chile). All observations, except of the first epoch, were performed with the ground-layer adaptive optics offered by the GALACSI module^50–52^ to improve the image quality. The first three were observed in YJH filters and the next four in YJHK_s_ filters. For the first three epochs we exposed for 3 × 60 s in the Y and J bands and 6 × 60 s in the H band. For the fourth epoch we also observe for 10 × 60 s in the K_s_ band. To account for the brightness evolution, we exposed for 10 × 100 s and 6 × 60 s for epochs 5 and 6. For the final epoch we also increased H-band exposure times to 10 × 60 s. For the HAWK-I observations we used offsets of (−115″, 115″) to place the target on the optimal detector chip.

The data used in our work have been reduced using the HAWK-I pipeline version 2.4.11 and the Reflex environment^53^. The data reduction included subtracting bias and flat fielding. The world coordinate system was calibrated against stars from Gaia.

##### MUSE observations and data reduction

We obtained four epochs of integral-field spectroscopy between 2022 August 24 and September 30 with MUSE^54^ at the ESO VLT. Each pointing has an approximately 1′ × 1′ field of view with spatial sampling of 0.2″ per pixel and covers the wavelength range from 4,700 to 9,300 Å with a spectral resolution of 1,800–3,600. All observations were performed with the ground-layer adaptive optics offered by the GALACSI module^50,52^ to improve the image quality. The integration time of each epoch was 1,800 s.

The data used in our work have been reduced using the MUSE pipeline version 2.8.7^55^ and the Reflex environment^53^. The data reduction included subtracting bias, flat fielding, wavelength calibration and flux calibration against spectrophotometric standard stars. Afterwards, we improved the sky subtraction with the Zurich Atmosphere Purge^56^ module in the ESO MUSE pipeline. The world coordinate system was calibrated against stars from Gaia.

#### Laser guide star adaptive optics imaging from Keck

The NIRC2 J-band observations consist of nine images in a five-point dither pattern based on a 2″ × 2″ grid size, to facilitate sky background subtraction. At the first dither location we obtained two images: one co-added 600 s exposure and one 200 s exposure. At the second location we obtained one 200 s exposure. At each of the third, fourth and fifth dither locations, we obtained two 200 s exposures. To correct for flat fielding and bias we acquired a set of ten bias frames (flat lamp off) and ten dome flat frames (flat lamp on). Sky background and dark current were removed as part of the sky subtraction, which utilized a different sky map for each dither position, created by median combining the frames from all other dither positions, excluding the current dither position. The final combined image was created by aligning each dither position to each of the others using the centroid of the brightest SN image (image A), and median combining. The reduction was carried out using custom Python scripts. The NIRC2 J-band data (Fig. 2) provides the highest-resolution (FWHM 0.086″) image in our dataset of the system where the four SN images are visible.

### Modelling of the lens galaxy

The Keck NIRC2 J-band image was used to model the lens galaxy in terms of its *θ*_E_, semi-minor to semi-major axis ratio *q* (or *ϵ*_e_ = 1 − *q*) and orientation angle *ϕ*. The mass profile used in our analysis is a singular isothermal ellipsoid^26,27^:

1 $$\kappa (x,y)=\frac{{\theta }_{{{{\rm{E}}}}}}{2\sqrt{q{x}^{2}+{y}^{2}/q}},$$

where *κ* corresponds to the convergence (that is the dimensionless projected surface mass density) and the coordinates (*x*, *y*) are centred on the position of the lens centre and rotated anticlockwise by *ϕ*. The projected mass *M* inside an isodensity contour of the singular isothermal ellipsoid is given by^27^

2 $$M=\frac{{c}^{2}}{4G}\frac{{D}_{{{{\rm{s}}}}}{D}_{{{{\rm{l}}}}}}{{D}_{{{{\rm{ls}}}}}}{\theta }_{{{{\rm{E}}}}}^{2}.$$

To calculate *D*_l_, *D*_s_ and *D*_ls_, we assumed a flat *Λ* cold dark matter cosmology with *H*_0_ = 67.4 km s^−1^ Mpc^−1^ and *Ω*_M_ = 0.315 (ref. ^24^).

In addition to the lens mass model, we included light models for the lens galaxy and SN host galaxy in the form of elliptical Sérsic profiles:

3 $$I(R)={I}_{{{{\rm{e}}}}}\exp \left\{-{b}_{n}\left[{\left(\frac{R}{{R}_{{{{\rm{e}}}}}}\right)}^{1/n}-1\right]\right\},$$

where *I*_e_ is the intensity at the half-light radius *R*_e_, *b*_*n*_ = 1.9992*n* − 0.3271 (ref. ^57^) and

4 $$R\equiv \sqrt{{x}^{2}+{y}^{2}/{q}_{{{{\rm{S}}}}}^{2}},$$

with *q*_S_ the axis ratio of the Sérsic profile. The SN images were modelled as point sources. We used a Moffat PSF with power index 2.94 and FWHM 0.091″ to model the full image. We simultaneously reconstructed the lens mass model, SN images and surface brightness distributions of the lens and host galaxy. The lens mass model is constrained only by the positions of the lensed SN images and not by their fluxes, since the latter may be considerably affected by substructures, such as stars, in the lensing galaxy. Our model contains 13 nonlinear free parameters: *θ*_E_, *ϕ*, *q*, *x*, *y* for the lens mass model, *R*_e_, *n*, *ϕ*_S_, *q*_S_, *x*_S_, *y*_S_ for the lens light model and *x*_SN_, *y*_SN_ for the SN position in the source plane. Our results are obtained using lenstronomy (https://lenstronomy.readthedocs.io/en/latest/), an open-source Python package that uses forward modelling to reconstruct strong gravitational lenses^57^. The result of the fit and comparison with the observations is shown in Supplementary Fig. 2. As a cross-check, we also modelled the HST photometry data and redid the analysis with LENSMODEL^58,59^, which produced consistent results.

The resulting best-fit values for the lens mass and light profiles are summarized in Supplementary Table 2. Additionally, we derived the gravitational mass within the isodensity contour given by the critical line of the lens (with radius *θ*_E_ = 0.167″, corresponding to 0.628 kpc) to be *M* = (7.82 ± 0.06) × 10^9^ *M*_⊙_.

Supplementary Table 3 displays the observed time delays and individual fractional flux contributions from each SN image as detailed in Lightcurve fit, magnification and time-delay inference (*t*_obs_ and *f*_obs_), compared with the predictions from the lens model (*t*_mod_ and *f*_mod_). Here, time delays are given with respect to image A: for example, *t*_*i*_ ≡ *t*_*i*_ − *t*_A_. The observed fractional flux ratios are measured from the Keck J-band image after subtracting the lens galaxy light, and the model predictions, *f*_mod_, are computed from the magnifications predicted by the lens model, *f*_*i*_ ≡ *μ*_*i*_/∑_*j*_*μ*_*j*_. In addition to the uncertainties obtained from the J-band image analysis, we make a conservative error estimate by also including the scatter in *f*_obs_ and *f*_mod_ obtained when modelling the system using data from the HST optical filters F475W, F625W and F814W, as well as the Keck near-infrared J-band data. Using this approach, we also take into account possible error contributions from uncertainties in dust extinction, lens galaxy subtraction and lens mass modelling.

The observed individual image magnifications can be obtained from *f*_obs_ by multiplying the individual fractional fluxes by the total observed SN magnification of ${\mu }_{{{{\rm{obs}}}}}^{{{{\rm{tot}}}}}=24.3\pm 2.7$. The total lens model image magnification, ${\mu }_{{{{\rm{mod}}}}}^{{{{\rm{tot}}}}}$, is sensitive to the lens mass slope (which is unconstrained by the imaging data), such that flatter halos predicts a larger magnification. For an isothermal lens, ${\mu }_{{{{\rm{mod}}}}}^{{{{\rm{tot}}}}}=14.9\pm 0.9$, indicating a flatter halo profile (see also ref. ^8^). However, the predicted flux ratios remain approximately constant, which means that to first approximation we can multiply the derived *f*_mod_ by an arbitrary ${\mu }_{{{{\rm{mod}}}}}^{{{{\rm{tot}}}}}$. In contrast to the predicted individual fractional flux contributions, the observed flux is dominated by images A and C. To match the observed values, substructure lensing is needed to additionally magnify images A and C with factors of >4 and >2, respectively, compared with any additional substructure (de)magnifications of images B and D.

To check the impact of added macro lens model complexity, we have studied cases where the lens mass distribution is modelled with two matter components: one where the surface mass density follows the lens light distribution (with an arbitrary mass-to-light ratio, possibly interpreted as a baryonic mass component), and a second one introducing a dark matter halo with additional flexibility on density profiles. In spite of the added extra complexity, the quality of the fit to the SN image positions does not improve, and induces shifts in the predicted flux ratios only below 5%, that is, very small in comparison with the observed flux ratio anomalies.

We do not detect any time dependence in the flux ratios between the Keck and HST images, observed just a month after the lightcurve peak, 6 d apart, or any other anomalous variation in the unresolved ~80-d-long lightcurve shown in Fig. 3. Comparing the expected size of SN photosphere with the Einstein radii of compact objects in the lensing galaxy, we infer that if the discrepancy from the smooth lens model is caused by microlensing the deflectors must exceed 0.02 *M*_⊙_.

With the aim of distinguishing between lensing effects by stars or larger substructures, we investigated the upper limit of image splitting for the brightest image, A. We approximated the maximum Einstein radius of a large structure at the position of image A by putting an upper limit on the difference in the FWHM between PSF_A_ and PSF_BCD_. Using equation (2), we inferred a 95% confidence upper limit on the substructure’s mass within its Einstein radius. Combined with the lower mass limit derived from the lack of flux ratio variability, this constrains the mass of the substructure deflector in the line of sight to image A to 0.02 < *M*/*M*_⊙_ < 3 × 10^7^.

We conclude that, similarly to the case of iPTF16geu, a smooth lens density fails to explain the individual image magnitudes and additional substructure lensing is needed. Since the observed properties of lens systems to first order depend only on the integrated mass within the images and/or the surface mass density of the lens at the image positions or in the annulus between the images^60^, small-image-separation systems provide a unique probe of the central regions of gravitational lensing galaxies. The probability for lens substructures, such as stars, to accommodate the needed (de)magnifications for a range of lens density slopes will be investigated in a future lens modelling paper.

### Lens galaxy photometry

We retrieved science-ready co-added images from Pan-STARRS DR1^61^. Using a circular aperture with a radius of 1.1″, we obtain the following apparent magnitudes of the lens galaxy: *g* = 22.09 ± 0.09, *r* = 20.71 ± 0.02, *i* = 20.14 ± 0.02, *z* = 19.84 ± 0.02 and *y* = 19.63 ± 0.05 (all errors are of statistical nature). We model the spectral energy distribution with the software package Prospector version 1.1^62^. Prospector uses the Flexible Stellar Population Synthesis (FSPS) code^63^ to generate the underlying physical model and Python-FSPS^64^ to interface with FSPS in Python. The FSPS code also accounts for the contribution from the diffuse gas (for example, H ii regions) based on the Cloudy models from ref. ^65^. Furthermore, we assumed a Chabrier initial mass function^66^ and approximated the star-formation history by a linearly increasing star-formation history at early times followed by an exponential decline at late times (functional form *t* exp(−*t*/*τ*)). The model was attenuated with the ref. ^67^ model.

The best-fit galaxy model points to a moderately massive galaxy with stellar mass log *M*_★_/*M*_☉_ = 10.1 ± 0.3. The other parameters, such as star-formation rate, attenuation and age are poorly constrained and we report their values only for the sake of completeness: star-formation rate = $1{5}_{-15}^{+21}\,{M}_{\odot }\,{{{\rm{yr}}}}^{-1}$, $E{(B-V)}_{{{{\rm{star}}}}}=0.{6}_{-0.4}^{+0.1}$ mag, ${{{\rm{age}}}}=1.{6}_{-1.2}^{+5.4}\,{{{\rm{Gyr}}}}$. We acknowledge that the aperture might not encircle the entire galaxy and, therefore, we might underestimate the stellar mass of the lens. Changing the radius of the measurement aperture to 4″ increases the mass by ~0.3 dex. This large aperture also includes the contribution of the SN host galaxy and therefore overestimates the stellar mass of the lens galaxy. Nonetheless, this upper bound does not alter our conclusion about the compact nature of the lensing galaxy, relative to other lensing systems.

### Host galaxy photometry

Numerous studies have shown that the peak absolute magnitudes of type Ia supernovae depend on their host galaxy masses (see, for example, ref. ^25^). Although the host and the lens galaxy are well separated in the HST images, both galaxies have diffuse emission extending well beyond the separation of the two galaxies. To first order, we can remove the contribution of the lens by subtracting the lens images predicted by lenstronomy from the HST images. Using a circular aperture with a 1″ radius and appropriate zero points from HST, we measure for the host 22.31 ± 0.11, 21.81 ± 0.06 and 21.13 ± 0.06 mag in F475W, F625W and F814W, respectively. Fitting the spectral energy distribution with Prospector with the same assumptions as in the previous section gives a galaxy stellar mass of $\log {M}_{\star }/{M}_{\odot }=9.{6}_{-0.3}^{+0.4}$. A closer inspection of the subtracted HST images reveals that the 1″ aperture does not encircle the total emission of the host. Increasing the aperture radius to 1.5″ encircles almost the entire host galaxy (F475W = 21.80 ± 0.09, F652W = 21.38 ± 0.04, F814W = 20.74 ± 0.03), but it also includes non-negligible contribution from residuals of the lens galaxy. The galaxy mass increases marginally to $\log M/{M}_{\odot }=9.{7}_{-0.4}^{+0.5}$ but is consistent with the previous measurement. This suggests that a mass-step correction is not required. To obtain a more robust estimate of the galaxy mass, near-infrared observations after the SN has faded are required.

### Supplementary information

Supplementary Information Supplementary Figs. 1 and 2 and Tables 1–3.

## Data Availability

The reduced spectra and lightcurves used in the paper are available through the WISeREP repository^68^ at https://www.wiserep.org/object/21343. The raw VLT data are also available from the ESO Science Archive Facility, http://archive.eso.org/eso/eso_archive_main.html, program ID: 109.234A.

## Code availability

The data were analysed using the public codes SNTD (https://sntd.readthedocs.io/en/latest/) and lenstronomy (https://lenstronomy.readthedocs.io/en/latest/). The Keck AO J-band images and analysis software can be requested from the first author. The Liverpool image reduction software will be made available upon publication by K-Taggart et al.
