# Supplementary material for: Uncovering a population of gravitational lens galaxies with magnified standard candle SN Zwicky
Source: Nat Astron. 2023 Jun 12;7(9):1098–107. doi: 10.1038/s41550-023-01981-3 (PMC10509034; doi:10.1038/s41550-023-01981-3)
Supplement: Supplementary file 1 — Supplementary Figs. 1 and 2 and Tables 1–3. [file 41550_2023_1981_MOESM1_ESM.pdf]

nature astronomy

Article

https://doi.org/10.1038/s41550-023-01981-3

Uncovering a population of gravitational lens galaxies with magnified standard candle SN Zwicky

In the format provided by the authors and unedited

Supplementary Information

Tables S1, S2, S3

Figures S1, S2

UT Date	MJD (days)	Phase (days)	Telescope Instrument	R $(\lambda/\Delta\lambda)$	λ range (Å)	Exp. time (s)
2022-08-21.21	59812.21	+2.7	P60/SEDM	100	4000-9200	2250
2022-08-22.92	59813.92	+3.9	NOT/ALFOSC	360	3700-9200	2400
2022-08-23.28	59814.28	+4.2	Keck/LRIS	820	3200 - 10200	600
2022-09-30.03	59852.03	+32.1	VLT/MUSE	1800 - 3800	4700-9300	1800

Table Supplementary 1: **Spectroscopic observations of SN Zwicky used for classification and redshift measurements.**

	$\theta_{\rm E}$ [$''$]	ϕ [°]	q	$R_{\rm e}$	n
Lens mass	0.1670 ± 0.0006	68.87 ± 0.15	0.653 ± 0.010	-	-
Lens light	-	68.11 ± 0.74	0.661 ± 0.008	1.74 ± 0.15	2.73 ± 0.10

Table Supplementary 2: **Summary of lens mass and light constraints from the Keck J-band data (Einstein radius $\theta_{\rm E}$, orientation angle ϕ, axis ratio q, half-light radius $R_{\rm e}$, and Sérsic index n). Note the agreement between the lens mass and light distributions.**

Image	$\Delta t_{\rm obs}$ [days]	$\Delta t_{\rm mod}$ [days]	$f_{\rm obs}$	$f_{\rm mod}$	$f_{\rm fit}$
A	-	-	0.367 ± 0.013	0.150 ± 0.004	0.376 ± 0.084
B	-0.4 ± 2.9	-0.51 ± 0.02	0.154 ± 0.005	0.285 ± 0.003	0.149 ± 0.060
C	-0.1 ± 2.3	-0.27 ± 0.01	0.304 ± 0.011	0.257 ± 0.004	0.300 ± 0.072
D	-0.1 ± 2.7	-0.44 ± 0.02	0.175 ± 0.005	0.309 ± 0.004	0.174 ± 0.065

Table Supplementary 3: **Observed arrival time differences (derived from the light curve) and individual fractional flux contributions from each SN image, compared to the ones predicted by the lens model and the lightcurve modelling. The discrepancy between the individual flux contributions shows that a smooth lens model cannot explain the observed SN image fluxes.**

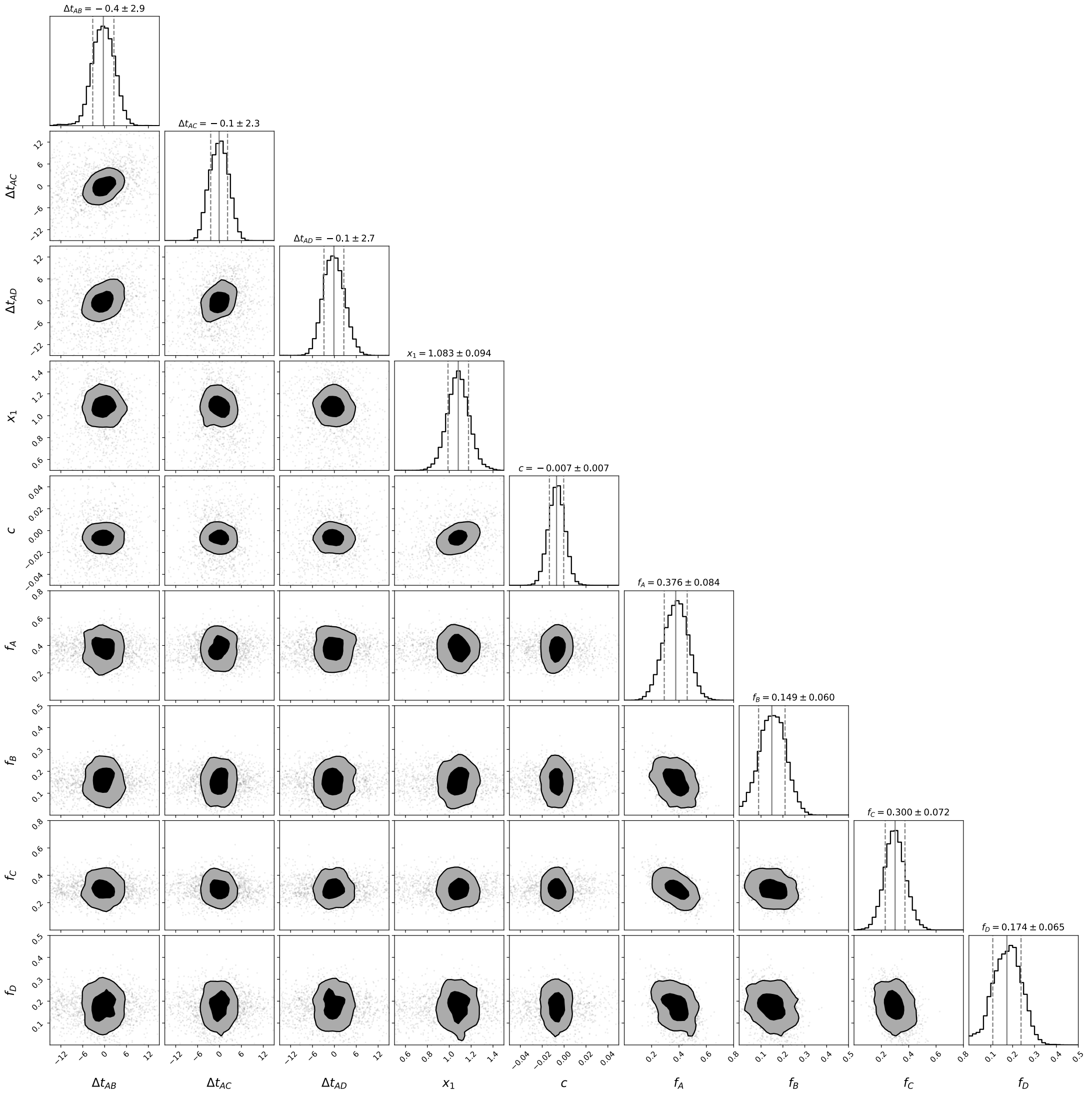

Figure Supplementary 1: **Corner plot showing the posterior distribution for the relative time-delay of the individual images B, C, and D to A,the fitted supernova parameters x_1 and c, as well as the reconstructed flux ratios at peak from the fit with the relevant correlations between each parameter. The 1D plots show the mean value with the 1 sigma uncertainty. The 2D plots show the 1 and 2 sigma contours.**

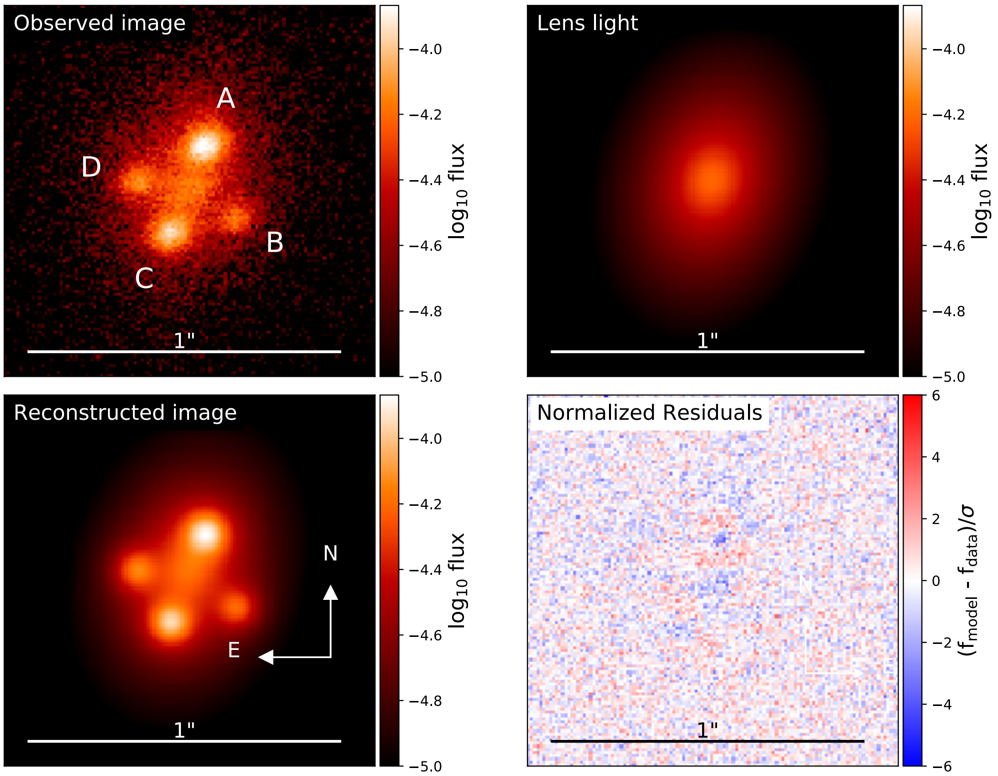

Figure Supplementary 2: **A comparison between the observed (data) image and the reconstructed (model) image for the Keck J-band photometry.** *Upper panel from left to right:* **The observed image and the reconstructed lens galaxy light intensity.** *Lower panel from left to right:* **The total reconstructed image (convolved with the PSF of the instrument, but without background or Poisson noise) and the normalized residuals.**
